# Establishing the distribution of cerebrovascular resistance using computational fluid dynamics and 4D flow MRI

**DOI:** 10.1038/s41598-024-65431-4

**Published:** 2024-06-25

**Authors:** Axel Vikström, Petter Holmlund, Madelene Holmgren, Anders Wåhlin, Laleh Zarrinkoob, Jan Malm, Anders Eklund

**Affiliations:** 1 Department of Diagnostics and Intervention, Biomedical Engineering and Radiation Physics, Umeå University, 901 87 Umeå, Sweden; 2 Department of Clinical Science, Neurosciences, Umeå University, Umeå, Sweden; 3 Umeå Center for Functional Brain Imaging, Umeå University, Umeå, Sweden; 4 Department of Applied Physics and Electronics, Umeå University, Umeå, Sweden; 5 Department of Diagnostics and Intervention, Surgical and Perioperative Sciences, Umeå University, Umeå, Sweden

**Keywords:** Carotid stenosis, Cerebrovascular resistance, Computational fluid dynamics, Peripheral cerebral territories, Stroke, Fluid dynamics, Carotid artery disease, Cardiovascular diseases, Vascular diseases, Stroke

## Abstract

Cerebrovascular resistance (CVR) regulates blood flow in the brain, but little is known about the vascular resistances of the individual cerebral territories. We present a method to calculate these resistances and investigate how CVR varies in the hemodynamically disturbed brain. We included 48 patients with stroke/TIA (29 with symptomatic carotid stenosis). By combining flow rate (4D flow MRI) and structural computed tomography angiography (CTA) data with computational fluid dynamics (CFD) we computed the perfusion pressures out from the circle of Willis, with which CVR of the MCA, ACA, and PCA territories was estimated. 56 controls were included for comparison of total CVR (tCVR). CVR were 33.8 ± 10.5, 59.0 ± 30.6, and 77.8 ± 21.3 mmHg s/ml for the MCA, ACA, and PCA territories. We found no differences in tCVR between patients, 9.3 ± 1.9 mmHg s/ml, and controls, 9.3 ± 2.0 mmHg s/ml (p = 0.88), nor in territorial CVR in the carotid stenosis patients between ipsilateral and contralateral hemispheres. Territorial resistance associated inversely to territorial brain volume (p < 0.001). These resistances may work as reference values when modelling blood flow in the circle of Willis, and the method can be used when there is need for subject-specific analysis.

## Introduction

A balanced blood flow distribution throughout the brain is crucial for maintaining adequate cerebral function. Cerebral blood flow distribution is intimately connected to the blood pressure driving the flow (i.e., the perfusion pressure) and the flow resistance of the cerebral arteries, known as cerebrovascular resistance (CVR). The cerebral autoregulation regulates CVR by varying the size of the diameters of the cerebral small arteries and arterioles, ensuring stable flow during changes in perfusion pressure^1^. Simultaneously, variations in metabolic demands induce changes in CVR through neurovascular coupling to secure a sufficient blood flow^2,3^. Therefore, deviations in CVR may reflect disturbances or depletion of these regulatory processes. Additionally, impaired vascular structure and function has been found to be increasingly important in the development of cognitive diseases^4–8^, making CVR and its distribution a potential screening tool in early cognitive decline^9^. Lastly, the vascular resistance of the different cerebral territories plays an essential role in the mathematical modelling and prediction of cerebral blood flow^10,11^. CVR is mostly estimated globally^12,13^, usually due to the simplicity of measuring total cerebral inflow and mean arterial pressure, as opposed to the local flow rates and perfusion pressures needed to determine CVR of each vascular territory. Knowing these territorial resistances would not only improve our understanding of physiology and pathophysiology, but could also be used for predictions of blood flow and perfusion pressure during planned interventions such as medical or surgical treatments^14^.

CVR can be computed from measurements of blood flow and perfusion pressure through an Ohm’s law equivalent^11^. The global measure of CVR, total CVR (tCVR), represents the vascular resistance against the flow from the cervical arteries to the parenchyma. It can be determined by the difference in mean arterial pressure (MAP) and intracranial pressure (ICP) divided by total cerebral blood flow (tCBF)^15^. A majority of the tCVR comes from the peripheral vessels in the territories supplied by the major cerebral arteries extending from the circle of Willis; the middle, anterior, and posterior cerebral arteries (MCA, ACA, PCA)^16,17^. CVR of these territories, territorial CVR, can be determined through the difference in the perfusion pressure and ICP divided by the flow in each exiting artery of the circle of Willis. Previous estimations of territorial CVR have been made with assumptions on arterial pressure drops and flow distribution in the branches of the circle of Willis^18,19^, the latter often approximated from territorial brain volume. Furthermore, these types of estimations are often reliant on literature data and thus not subject-specific data. Four-dimensional phase-contrast magnetic resonance imaging (4D flow MRI) allows for simultaneous flow measurements in the vessels of the entire brain^20^, eliminating the need for flow distribution assumptions. To accurately estimate the territorial resistance, the measured flow rates to each territory should be accompanied by their corresponding perfusion pressures, which can be determined with computational fluid dynamics (CFD) modelling of the circle of Willis^21^. Consequently, this allows for the assessment of each territorial resistance in a subject-specific manner while utilizing accurate flow rate measurements and accounting for pressure drops in the circle of Willis.

In this study, we aim to establish the distribution of CVR in a group of patients with a transient ischemic attack or stroke, with and without significant carotid stenoses. With this patient group we examine possible lateral differences in CVR from hemodynamic disturbances induced by carotid stenoses. A control group of elderly was included for comparison of tCVR. As a secondary analysis we examine the relationship between CVR and brain volume, in total as well as intra- and inter-territorially, to test the feasibility of using brain volume distribution as a proxy for resistance distribution.

## Material and methods

### Overview of approach

**Figure 1:**
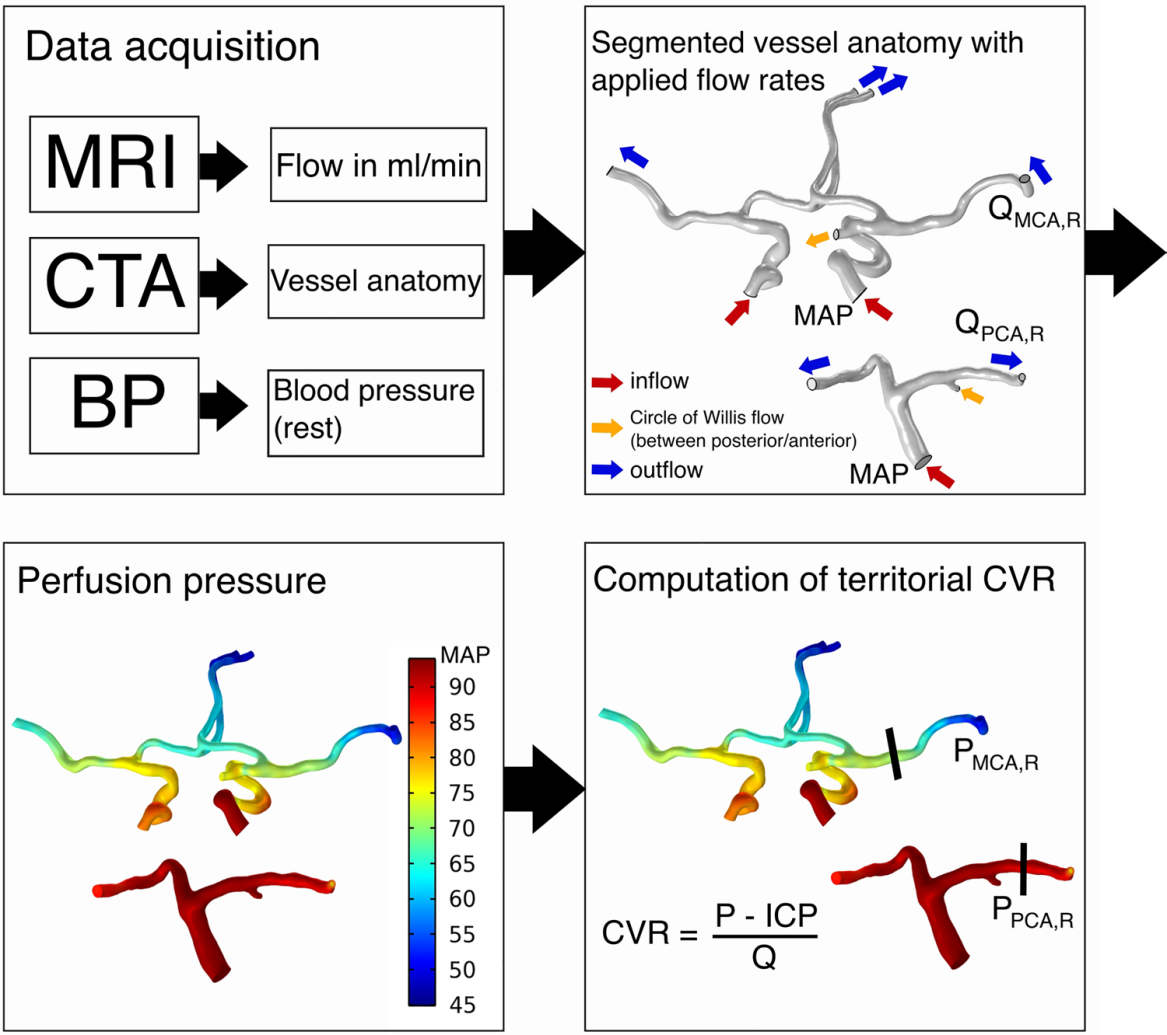
Overview of our method for determining territorial CVR. Computational domains of the anterior and posterior parts of the circle of Willis are segmented from CTA data, and to them we apply the measured flow rates and MAP. This subject has a right posterior communicating artery, connecting the anterior and posterior parts of the circle of Willis (flow indicated by orange arrows). CFD is then used to compute the perfusion pressure in the circle of Willis given these conditions. The resulting perfusion pressure, in combination with the flow rates along each outflow branch (blue arrows), allows for computing territorial CVR, i.e. the lumped resistance downstream of each cerebral artery.

Vascular resistances of each cerebral arterial territory were computed on a subject-specific level (Fig. 1). The arterial tree of each patient was segmented from computed tomography angiography (CTA) data. The flow rate in each artery was acquired from the 4D flow MRI data. With the segmentation and flow rates, CFD computations estimate the perfusion pressure at each branch of the circle of Willis. The computed perfusion pressures together with the flow rate to each territory allowed for an assessment of CVR in the cerebral regions fed by the ACA, MCA, and PCA.

### Subjects

**Figure 2:**
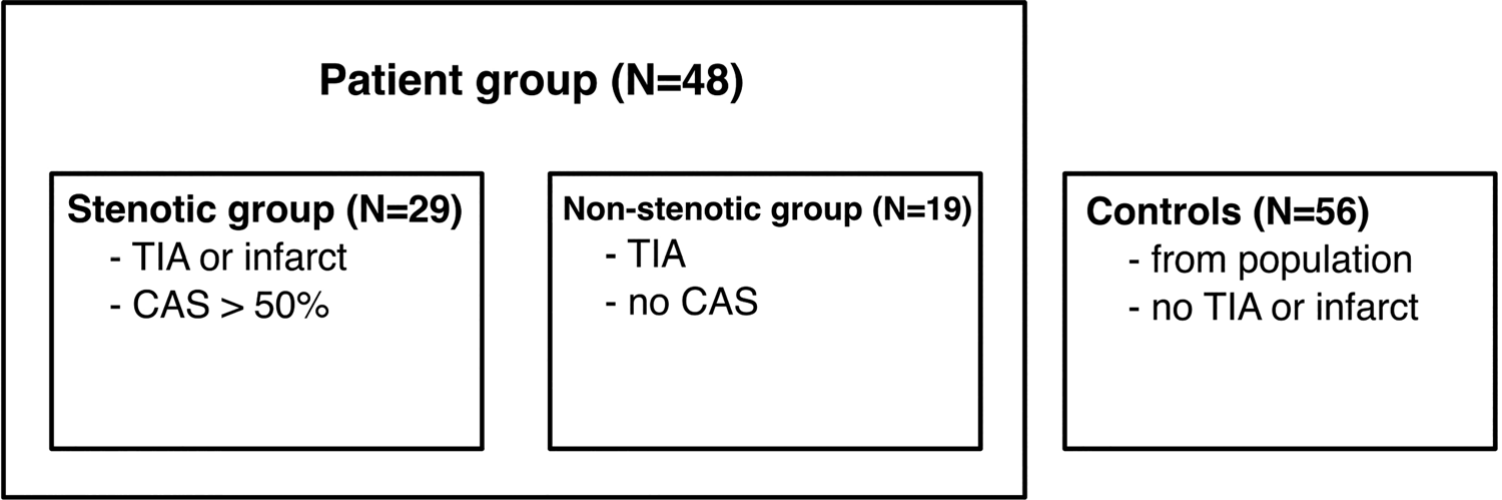
Schematic of the subject groups. *CAS* carotid artery stenosis.

The study population consisted of a patient group with two subgroups and a control group (Fig. 2). The patient subgroups originated from the stroke unit at Umeå University Hospital between 2012 and 2015. 29 patients were included in the stenotic group. They had a transient ischemic attack (TIA) or cerebral infarct and a symptomatic carotid stenosis ≥ 50%, with or without a non-symptomatic contralateral stenosis. Ten of these patients presented an ipsilateral stenosis of degree 50–69%, whereas the remaining 19 had a degree ≥ 70%. Nine patients had a non-symptomatic contralateral stenosis. The non-stenotic group consisted of 19 patients with TIA, without stenoses. The North American Symptomatic Carotid Endarterectomy Trial (NASCET) method was used for grading the stenosis^22^. The side with the symptomatic stenosis was defined as the ipsilateral side, with the opposite being the contralateral side. No patient had any intracranial stenoses. A control group was recruited from the Swedish population registry (N = 61)^23^. It consisted of elderly, irrespective of previous disease, but those with previous TIA or stroke were excluded (N = 3). MRI was incomplete for two controls. Territorial CVR could not be estimated in the control group due to the lack of CTA (not motivated in healthy) but 4D flow data and mean arterial pressure (MAP) were available, allowing for computation and comparison of tCVR. Full characteristics are listed in Table 1.

**Table 1:** Characteristics of the patients and controls.

	Stenotic group (N = 29, 22 men)	Non-stenotic group (N = 19, 13 men)	All patients (N = 48, 35 men)	Controls (N = 56, 36 men)
Age (years)	**73 ± 6**^†^	**67 ± 7**^†^	71 ± 7*	74 ± 4*
Stenosis degree (%)	75 ± 12 (ipsilateral) 67 ± 10 (contralateral, N = 9)	N/A	N/A	N/A
mRS score	0 (0–1)	0 (0–0)	0 (0–1)	N/A
NIHSS score	1 (0–6)	0 (0–3)	1 (0–6)	N/A
SBP (mmHg)	137 ± 17	132 ± 19	135 ± 18*	142 ± 19*
DBP (mmHg)	70 ± 10	74 ± 12	72 ± 11*	81 ± 10*
MAP (mmHg)	92 ± 11	94 ± 13	93 ± 12*	102 ± 12*
Heart rate (bpm)	65 ± 11	69 ± 10	67 ± 11	68 ± 11
Hypertension (N)	22 (76%)	11 (58%)	33 (69%)	31 (55%)
Hyperlipidemia (N)	16 (55%)	6 (32%)	22 (46%)*	12 (21%)*
Diabetes mellitus (N)	7 (24%)	4 (21%)	11 (23%)	6 (11%)
Ever smoker (N)	19 (66%)	9 (47%)	28 (58%)	36 (64%)

Significant difference was tested with Student’s t-test for continuous variables and chi-squared test for categorical variables between the patient subgroups (^†^) as well as between all patients and the controls (*), where significance (p < 0.05) is highlighted in bold. *mRS* modified Rankin Score, *NIHSS* National Institutes of Health Stroke Scale, *SBP* systolic blood pressure, *DBP* diastolic blood pressure, *MAP* mean arterial pressure.

The study was approved by the ethical review board of Umeå University (Dnr: 2011-440-31 M) and the Swedish Ethical Review Authority (Dnr: 2019-05909) and was performed in accordance with the guidelines of the Declaration of Helsinki. All participants were given oral and written information about the study and written consent was obtained from all participants.

### Imaging

For each patient, 4D flow MRI and CTA data with full brain coverage was available. The median number of days between scans was 4 (IQR 2–8). 92% (44/48) of patients had their MRI scan within two weeks of onset/CTA. A 3 T scanner with a 32-channel head coil (GE Discovery MR 750, Milwaukee, WI, USA) was used for the MRI scans providing flow rates in the cerebral vessels, utilizing a balanced 5-point phase contrast vastly undersampled isotropic projection reconstruction (PC-VIPR) sequence^24,25^. The velocity encoding was 110 cm/s. Reconstructed MRI voxel size was 0.7 × 0.7 × 0.7 mm^3^. The CTA images used to segment patient-specific vessel geometries were obtained from different hospitals, with reconstructed slice thickness ranging between 0.3 and 0.625 mm. More information regarding settings for both imaging modes can be seen in the study of Holmgren et al.^21^. The reason for using CTA for vessel geometry was partly due to the superior resolution, but also due to the fact that CTA more reliably visualizes the vasculature compared to MRI methods for visualization, which are blood flow velocity dependent (especially in vessels with low blood flow).

### Vessel geometries

**Figure 3:**
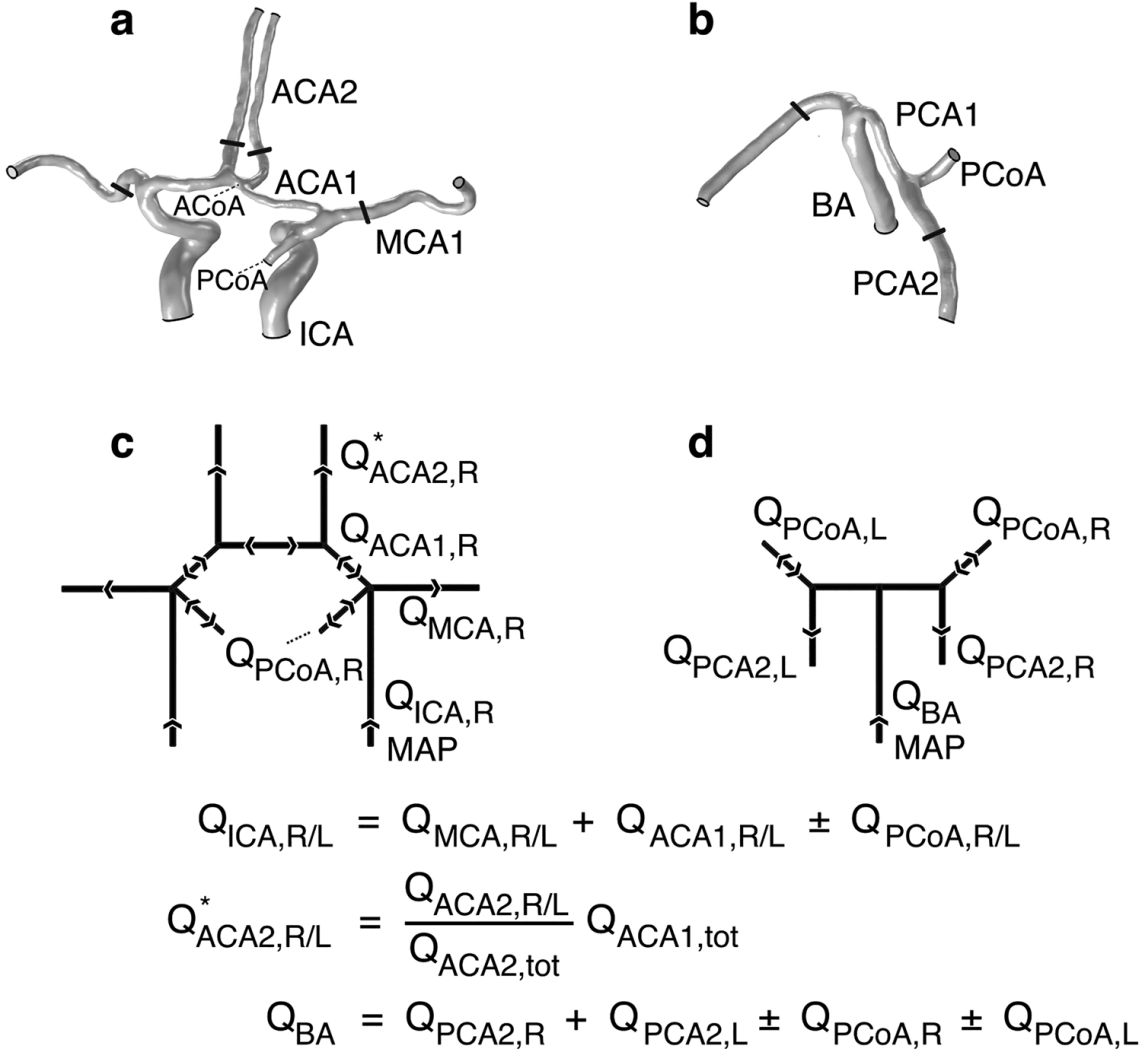
Examples of (**a**) anterior and (**b**) posterior geometries (not to scale) from the same subject and cuts from which the computed pressure was read. In the bottom row are general schematic illustrations of the (**c**) anterior and (**d**) posterior boundary conditions used for the CFD. The flow rates were assessed by 4D flow MRI. Note the variability in sign for ${Q}_{PCoA}$, introduced as an additional inlet requires a reduction of the corresponding ${Q}_{ICA}$ in anterior models and ${Q}_{BA}$ in posterior models to preserve mass conservation.

The vessel geometries were semi-automatically segmented from the CTA data, performed with Synopsys’ Simpleware™ software (ScanIP P-2019.09; Synopsys, Inc., Mountain View, USA). The segmentation included the complete circle of Willis with the internal carotid arteries (ICA), the basilar artery (BA), the middle cerebral arteries (MCA1), proximal and distal posterior cerebral arteries (PCA1/PCA2), proximal and distal anterior cerebral arteries (ACA1/ACA2), and the anterior and posterior communicating arteries (ACoA and PCoA). The segmentation process was carried out as previously described^21^. The circle of Willis geometries were separated into an anterior and posterior part to ensure the correct flows in the subsequent simulations and to avoid possible underdetermination of the flow equations introduced by the circular structure of the circle of Willis. Examples of each type are shown in Fig. 3a and b.

AV segmented all vascular trees of the non-stenotic group, as well as one complete and 20 posterior geometries from the stenotic group. MH segmented 28 anterior geometries from the stenotic group and the remaining 8 posterior geometries were segmented by PH. AV re-segmented 10 geometries from the stenotic group to examine inter-rater agreement through intraclass correlation.

### Flow rates

Average flow rates for each ICA, MCA1, ACA1, ACA2, BA, PCA1, PCA2, and PCoA were obtained from the 4D flow MRI data using MATLAB (R2022b, MathWorks, Natick, MA). The acquisiton is made with a post-processing method that averages consecutive flow waveforms^26,27^, see Fig. 2 in the review by Wåhlin et al.^28^. The average flow rates to be used for boundary conditions in the simulation were assessed by averaging 15 cross-sections of each artery segmented and calculated from the 4D flow data. Thus, the vessel geometry and the flow rate assessment were performed separately and did therefore not require co-registration.

### CFD simulations

Stationary CFD models solving the Navier–Stokes equations were constructed using the CFD module in COMSOL Multiphysics® (COMSOL Multiphysics®, version 5.4, www.comsol.com, COMSOL AB, Stockholm, Sweden). The Navier–Stokes equations are the governing equations for fluid flow and relates total fluid acceleration to the forces acting upon it. We used the Navier–Stokes equations for incompressible fluids:

1 $$\rho \left(\frac{\partial {\varvec{u}}}{\partial t}+{\varvec{u}}\cdot \nabla {\varvec{u}}\right)=-\nabla p+\mu {\nabla }^{2}{\varvec{u}},$$

$$\nabla \cdot {\varvec{u}}=0,$$

where ${\varvec{u}}$ is the velocity field, $p$ the pressure, $\rho$ the density, and $\mu$ the viscosity of the fluid. Blood flow was assumed to be laminar with blood defined as an incompressible Newtonian fluid with a density and viscosity of $\rho$ = 1060 kg/m^3^ and $\mu$ = 3.45 mPa s^29,30^. Rigid walls were used, which is sufficient when studying mean flows and pressures. Meshes were automatically generated in ScanIP, with refinement in the circle of Willis as well as the closest segments of the cerebral arteries and ICAs. In the refinement area, the element size was set to equal the interpolated voxel size. Otherwise, the element size was increased by a factor of 1.5.

The left and right ICA as well as the BA boundaries were defined as inlets, whereas the left and right MCA1, ACA2, and PCA2 boundaries were defined as outlets. Any possible PCoA boundary would be defined as an outlet or inlet depending on the direction of the flow. Consequently, flow through a PCoA from the anterior to the posterior circulation implied an outlet boundary in the anterior model and an inlet boundary in the corresponding posterior model, whereas a reversed flow direction would imply the opposite. COMSOL’s built-in boundary condition for fully developed flow was enabled for inlets and outlets, applying laminar parabolic flow rate profiles. The vessel walls were given a no-slip condition.

To achieve mass conservation, in addition to correct flow through the circle of Willis, the inlet flow rates were based on summed flow rates of arteries in the circle of Willis. In anterior models, the inlets of the left and right ICA were set to the sum of each respective MCA1, ACA1, and possible PCoA flow rate. In posterior models, the BA inlet flow rate was set to the sum of all PCA2 and PCoA outflows. As an additional measure, an adjustment of the ACA2 outlets was applied such that the ACA1 flow rates were correct according to the 4D flow MRI measurements. See Fig. 3c and d for schematics of the boundary conditions.

MAP was assumed as reference pressure in the contralateral ICA and the BA, and was applied as a pressure point at their boundaries. In the non-stenotic group, lacking significant stenoses, MAP was assumed in the ICA with the largest flow rate. For subjects in the stenotic group with a contralateral stenosis ≥ 50%, the pressure at the contralateral ICA was found by subtracting the pressure drop across the stenosis from the MAP, where the pressure drop was computed in a separate CFD simulation of the stenosis^14^.

### Computing the territorial resistance

A finished simulation generated a steady state solution of the average pressure distribution in the geometry. The average perfusion pressures in the MCA1, ACA2, and PCA2 were obtained at cross-sectional cut planes placed perpendicular to the flow direction, positioned five millimeters from the bifurcations of the outflow arteries (of the circle of Willis, as seen in Fig. 3a,b). When the PCoA was missing, the PCA cut plane was positioned five millimeters from the BA bifurcation. In case of double of any cerebral artery, e.g. two left MCA1, resistance was estimated as the parallel resistance between the two (two MCA1, N = 1; two ACA2, N = 4).

Having determined the pressure *P*_*terr*_ at the beginning of each major cerebral artery we computed the total downstream resistance, or territorial resistance, *R* as

2 $$R=\frac{{P}_{terr}-ICP}{{Q}_{terr}}= \frac{\Delta P}{{Q}_{terr}},$$

where *Q*_*terr*_ is the flow rate through the vessel and *ICP* the intracranial pressure which is the counter-pressure in the brain tissue (in line with the definition of cerebral perfusion pressure^31^). Similarly, total CVR (tCVR) was computed as

3 $$tCVR=\frac{MAP-ICP}{{Q}_{total}}$$

where ${Q}_{total}$ is the total cerebral inflow, i.e. the sum of the ICA and BA flow rates. For ICP, we used a reference value in healthy elderly of 11.6 mmHg^32^.

### Computing brain volumes

Brain volumes were computed with FreeSurfer 7.2.0 (https://surfer.nmr.mgh.harvard.edu/) for comparisons against CVR. In FreeSurfer, the regions of the brain are automatically segmented from T1-weighted images into grey matter, white matter, and cerebrospinal fluid^33^. Grey and white matter volumes of each territory were included. Which regions that belonged to each of the MCA, ACA, and PCA territories was determined from the stratification made by Tatu et al.^34^. By summing up the volumes of the regions (acquired from FreeSurfer) in each respective territory, the volumes of the vascular territories were computed. One subject lacked a T1 image, but the FreeSurfer algorithm successfully segmented the brain regions in all but one (where a consistent delineation was not possible) of the remaining subjects. Results were visually inspected for accuracy using FreeView (version 3.0, the General Hospital Corporation, Boston, MA).

### Statistical analysis

Firstly, we wanted to compare tCVR of the controls to that of the patients to assess global response of CVR in the event of hemodynamic disturbance. Secondly, territorial CVR within the patient group was compared between the clinically determined ipsilateral and contralateral hemispheres. Both ipsilateral and contralateral resistances were, in turn, compared to all territorial resistances of the non-stenotic group (averaged over hemispheres). A total of 279 territorial resistances were calculated. One patient lacked one PCA, another lacked one MCA, and 7 PCA territories were excluded due to posterior stenoses. Lastly, for a more perfusion-based comparison, an analysis was performed where hemispheres were separated by flow rates instead of stenosis degree, where an ICA flow rate below 160 ml/min was used to classify hemodynamic disturbance^35^. Territorial CVR of hemispheres classed as hemodynamically disturbed were compared pairwise to the corresponding territories of the patients’ opposite hemispheres.

We also wanted to examine the correlation between CVR and brain volume, as territorial CVR previously have been estimated on flow rate assumptions such as distribution by brain volume. Both inter- and intra-territorial relationships between resistance and brain volume were investigated. Total and territorial brain volumes could be computed for 46 patients. As resistance is expected to relate inversely to volume it is appropriate to utilize the reciprocal of the vascular resistance, vascular conductance^36^, when analyzing this relationship.

All values are presented as mean with standard deviation. Lilliefors’ goodness-of-fit test was used for normality testing. For paired as well as unpaired tests of significance, Student’s t-test was used. Correlation between total conductance and volume was examined with Pearson correlation coefficient. Relations between territorial conductance and volume were examined with a linear mixed model based on restricted maximum likelihood, accounting for hemisphere as well as there being multiple samples from each subject. In the linear mixed model, parameters were set as; conductance as dependent variable, hemisphere (ipsilateral/contralateral/non-stenotic) as fixed factor, volume as covariate, and subject ID as random effect. P < 0.05 was the threshold for statistical significance. Intra- and inter-territorial comparisons of CVR were Bonferroni corrected by 18 tests, resulting in P < 0.003 for significance. The statistical analysis was carried out in MATLAB and jamovi (2.3.28.0, The jamovi project, Sydney, Australia). Intraclass correlation coefficient (ICC) analysis was performed to compare the segmentations between two different raters using two-way random effects, absolute agreement, single rater/measurement. All resistances for 10 subjects were used in the comparison.

## Results

The inter-rater agreement for segmentation of the arterial tree was excellent, as shown by the ICC: r = 0.99, CI [0.980–0.994], p < 0.001. The corresponding CI for the MCA, ACA, and PCA were all within [0.930–0.998].

### Comparing tCVR among patients and controls

**Figure 4:**
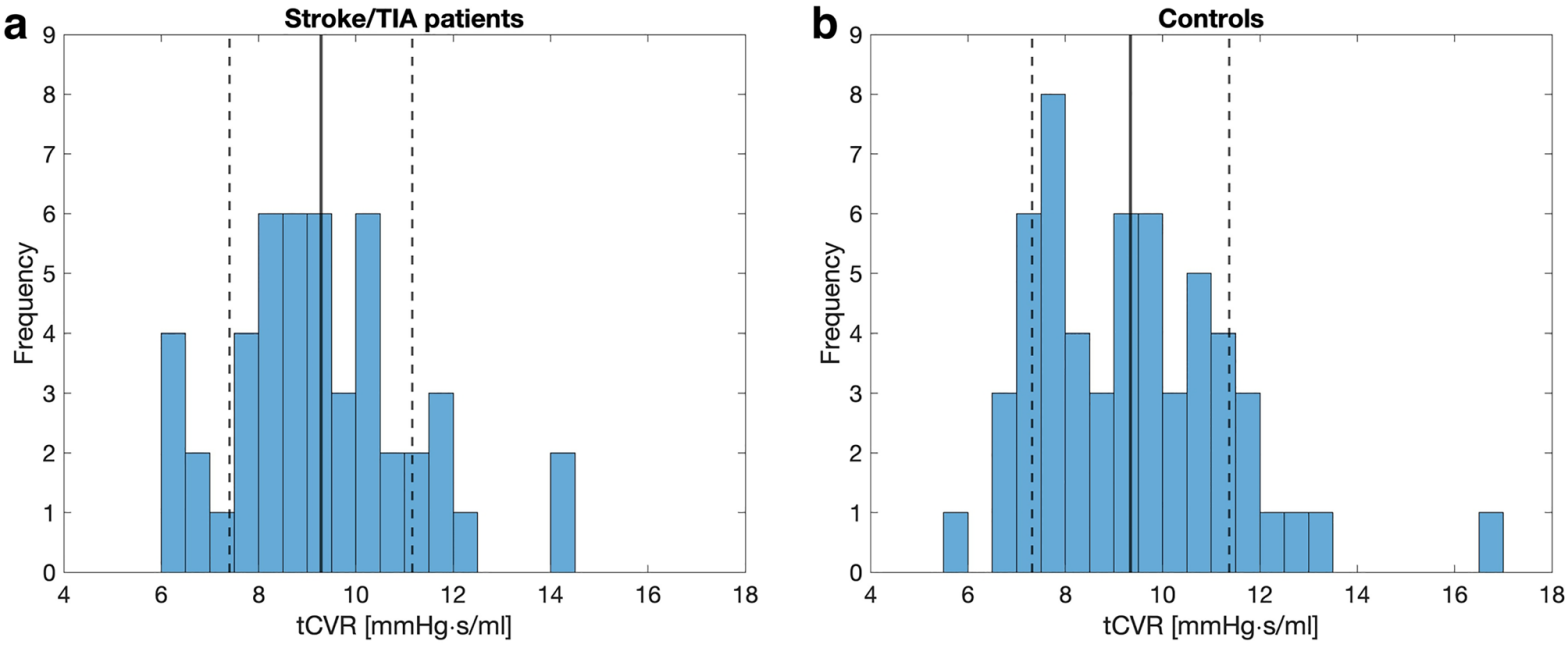
Distribution of tCVR for (**a**) the patient group and (**b**) the controls with marked mean and standard deviation, between which no significant difference was found with unpaired Student’s t-test (p = 0.88).

The average tCVR was 9.3 ± 1.9 mmHg s/ml for all patients (N = 48). It was 9.3 ± 1.7 mmHg s/ml for the stenotic group (N = 29) versus 9.3 ± 2.1 mmHg s/ml for the non-stenotic group (N = 19) (p = 0.91). The controls (N = 56) had a mean tCVR of 9.3 ± 2.0 mmHg s/ml, which was not different compared to the patient group (p = 0.88). tCVR distribution among the patients and controls are given in Fig. 4a and b, respectively. Total cerebral inflow for the controls was 9.9 ± 1.4 ml/s, significantly higher (p < 0.01) compared to the patient group inflow of 9.0 ± 1.4 ml/s.

### Distribution of territorial CVR

**Figure 5:**
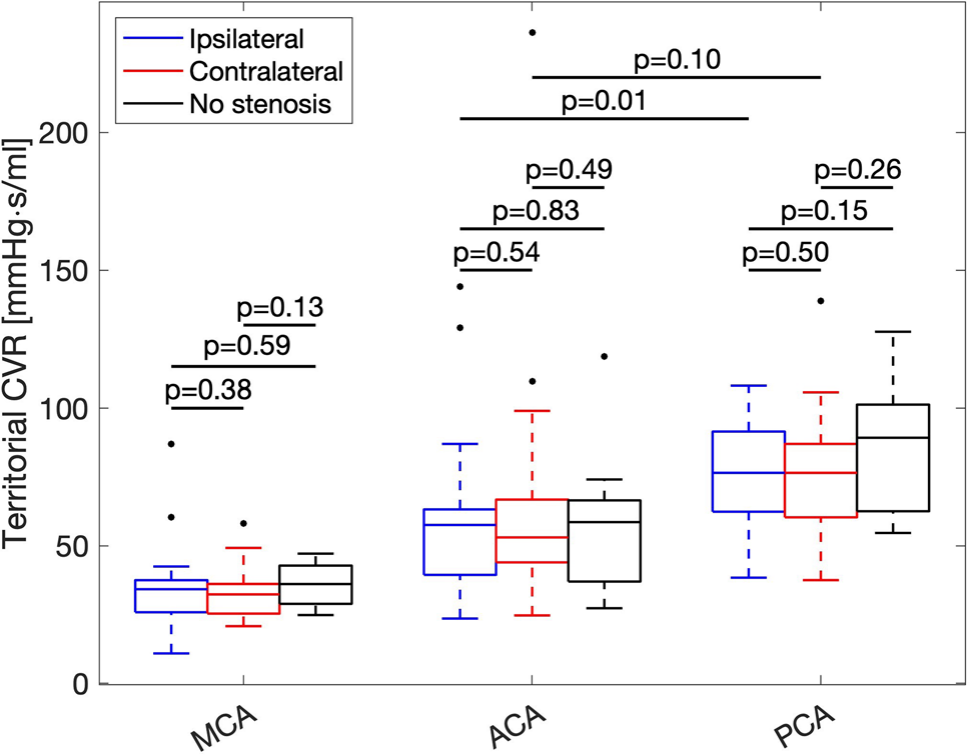
Distributions and comparisons of territorial CVR within and between territories based on hemispheres. With Student’s t-test, there were no differences between hemispheres within each territory (all p > 0.05) but significant difference between most territories except those highlighted (all p < 10^–4^, p = 0.01 insignificant if Bonferroni-corrected).

No differences in CVR were found within territories when comparing the ipsilateral and contralateral hemispheres of the stenotic group, nor when comparing each of them with the non-stenotic group’s averaged hemispheres (all p > 0.05, Fig. 5). Significant differences in CVR were observed between territories (p < 10^–4^, Fig. 5), except between the contralateral ACA and PCA (p = 0.10) as well as ipsilateral ACA and PCA (p = 0.01, insignificant with Bonferroni-correction). Including values from all patients, CVR were 33.8 ± 10.5, 59.0 ± 30.6, and 77.8 ± 21.3 mmHg s/ml for the MCA, ACA, and PCA territories. All numerical values of territorial CVR are given in Table 2. Identifying hemispheres below a threshold ICA feeding flow rate of 160 ml/min and comparing the territories to the corresponding territories on the opposite hemisphere gave no significant difference in territorial CVR for any territory.

**Table 2:** Mean resistances for the territories.

	All (mmHg s/ml)	Ipsilateral (mmHg s/ml)	Contralateral (mmHg s/ml)	Non-stenotic group* (mmHg s/ml)
MCA	33.8 ± 10.5 (N = 77)	34.0 ± 13.7 (N = 29)	32.3 ± 8.2 (N = 29)	35.9 ± 7.5 (N = 19)
ACA	59.0 ± 30.6 (N = 77)	57.4 ± 26.9 (N = 29)	62.6 ± 38.8 (N = 29)	55.8 ± 21.1 (N = 19)
PCA	77.8 ± 21.3 (N = 71)	74.6 ± 20.8 (N = 27)	76.8 ± 20.9 (N = 27)	84.4 ± 22.5 (N = 17)

No significant differences in CVR between the hemispheres were found. *Note that territories are averaged over the hemispheres for the non-stenotic group.

### Relationship between resistance and brain volumes

**Figure 6:**
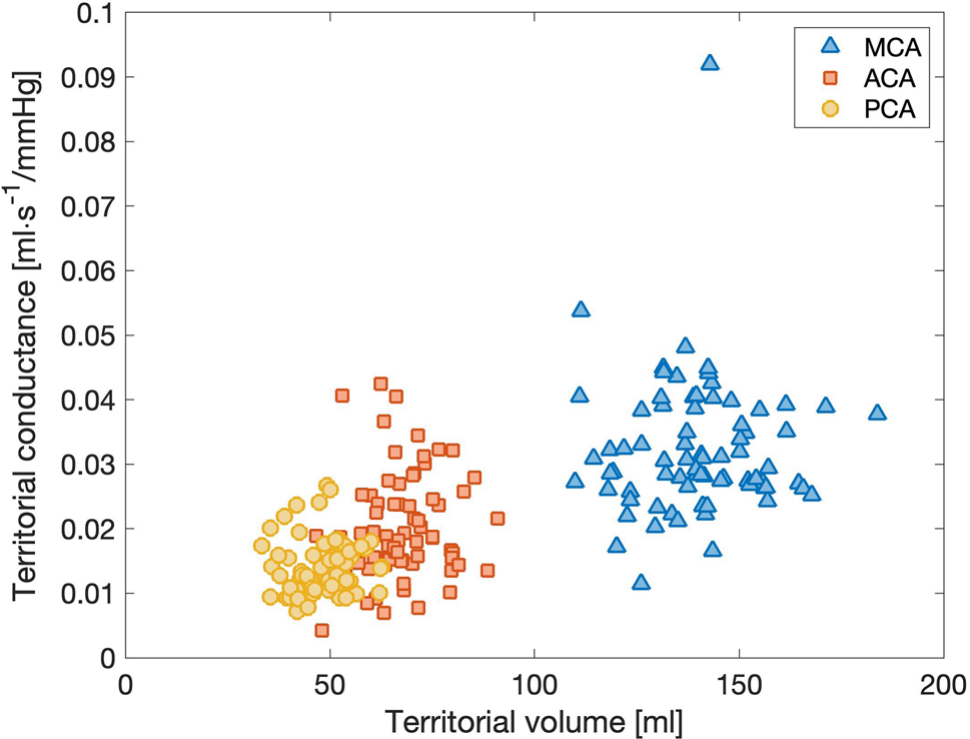
Territorial conductance and volume for the patients (N = 46). With the linear mixed model, there was an association between conductance and volume (p < 0.001). This was not true for each respective territory (MCA, p = 0.44; ACA, p = 0.54; PCA, p = 0.49). MCA outlier was mainly supplied by flow through the ACA1 and ACoA, resulting in a large drop in perfusion pressure and thus a low computed resistance.

Average total brain volume was 1065 ± 108 ml and correlated significantly to total conductance with Pearson correlation coefficient (N = 46, r = 0.37, p = 0.01). When analyzing territorial conductance and volumes with the linear mixed model we found that when all territories are included, volume associates to conductance (p < 0.001). The model estimates the association with an intercept of 0.0224 ml/mmHg s, 95% CI [0.0207, 0.0242], and volume-conductance slope of 1.84 × 10^–4^ 1/mmHg s, 95% CI [1.63 × 10^–4^, 2.04 × 10^–4^]. However, applying the model to each respective territory yielded no association between conductance and volume (MCA, p = 0.44; ACA, p = 0.54; PCA, p = 0.49). Distribution of territorial conductance and volume is illustrated in Fig. 6.

## Discussion

We present the territorial distribution of CVR for stroke/TIA patients with and without symptomatic carotid stenoses. With arterial anatomy from CTA and blood flow from 4D flow MRI data in combination with CFD pressure estimation, we propose a subject-specific method for determining territorial CVR without relying on flow distribution assumptions. There were differences in CVR between most of the MCA, ACA, and PCA territories. There was no difference between hemispheres in the stenotic group, nor any difference in tCVR between the patient and the control group. Our results suggest that CVR may be contained within fairly stable levels in these patients, despite significant carotid stenoses and diminished flow, i.e., the expected regional autoregulatory control had an unexpectedly low impact. For future work these resistances may work as reference values when modelling blood flow in the circle of Willis, and the method can be used when there is need for subject-specific analysis.

### Distribution of CVR

In the brain, regulatory systems such as autoregulation and neurovascular coupling utilize CVR to ensure a sufficient blood supply^1–3^. Thus, it is reasonable that hemodynamic disturbances such as carotid stenoses would impact CVR through e.g. dilation of arteries distal of a stenosis. Total CVR represents the relationship between the pressure drop from MAP in the cervical arteries to ICP in the parenchyma and the total blood supply feeding the brain. In our study, we found the patients to have a tCVR of 9.3 ± 1.7 mmHg s/ml and the controls 9.3 ± 2.0 mmHg s/ml, with comparable histograms (Fig. 4). This is slightly smaller compared to previous studies, where tCVR of patients with cerebrovascular disease has been reported as 9.8 ± 2.1^13^ and of healthy as 10.5 ± 2.1 mmHg s/ml^12^. The difference can largely be explained by the inclusion of ICP in our computations, which is substantial in the supine position^32^. Interestingly, we found no difference in tCVR between controls and the hemodynamically affected patients. This could potentially be explained by the stage in the disease development at which the patients were examined. Previous studies have shown that regulatory processes in the brain are impaired by carotid stenoses and stroke^37^. In addition, MAP was lower among patients compared to controls, in contrast to reports of elevated blood pressure for such patients prior to medication^38^. Medication may play a role in further inhibiting regulation to relieve blood pressure and therefore bring cerebrovascular properties back to normal.

We also did not find any differences in territorial CVR between patient hemispheres (Fig. 5, Table 2), despite the presence of carotid stenoses. Since it could be possible that contralateral stenoses could affect this comparison, we did a post-hoc analysis where patients with a contralateral stenosis were removed, and the results remained the same. Thus, there seems to be no difference in tCVR between hospitalized patients (with limited amount of symptoms indicated by low mRS and NIHSS scores) compared to controls, nor in territorial CVR between the patient hemispheres, suggesting that the presented distribution of CVR in patients (under treatment) reflects that of healthy elderly. It is possible that the CVR distribution may have differed at an earlier stage prior to the ischemic event and subsequent treatment, where the previously discussed adverse effects on autoregulation were not present. This was not possible to investigate in the current study, which motivates additional studies where the CVR distribution is assessed at different points in time over the cerebrovascular disease development.

When comparing the resistances of different territories, the resistances differed significantly, with a distribution of 34:59:78 mmHg s/ml for MCA:ACA:PCA (Table 2). An early and well referenced distribution of CVR was made by Hillen et al., in which the territorial resistances were estimated assuming inverse proportion to the mass irrigated by the vessel^18,39^. They reported a distribution of 30:60:40 mmHg s/ml. Stergiopulos et al.^40^, reported CVR downstream of ICA which, by assuming proportionality to the initial cross-sectional area of the cerebral arteries^41^, resulted in 45:64:83 mmHg s/ml. Another approach to find territorial CVR is to apply outflow conditions containing estimates of territorial CVR and updating them until the model re-creates a measured value, such as total cerebral blood flow and external carotid artery flow rate^19^. This verification-based approach yielded a distribution of 26:93:69 mmHg s/ml. A similar approach, where verification was made against arterial spin labeling data of perfusion amounts in the territories, yielded distributions 11:17:35 mmHg s/ml and 24:53:82 mmHg s/ml for two stenosis patients, as well as 9:24:47 mmHg s/ml for a young, healthy control^42^. Notably, the distributions from these methods vary not only in size, but also in relation among territories. There could be several reasons for this, such as small subject groups or the reliance upon flow rate assumptions, or reference data built upon such assumptions. We offer values from a subject-specific method without flow rate assumptions in conjunction with local perfusion pressures in the circle of Willis. Additionally, results were acquired from a set of subjects much larger than most medical CFD studies, making for a more rigorous assessment than previously done.

One of the most common ways to estimate CVR has been to divide the tCVR over the territories depending on their respective volumes. It is an attractive approach, as volumes are more widely accessible than exact flow rates. By determining total and territorial volumes of the patients, we were able to further investigate the relation between CVR and brain volume. The total and territorial brain volumes of our study are comparable to previous work^43,44^. Our results show a positive correlation between total conductance (i.e. negative correlation for tCVR) and total brain volume. Similarly, the linear mixed model showed that territorial conductance could be explained by territorial volume, which was expected but has not yet been shown. However, this could not be shown within the respective territories. We interpret these results as that the *distribution* of CVR over the territories can be assumed with brain volume or mass assumptions, but that a territorial volume cannot directly be converted into a territorial resistance due to larger inter-subject differences.

### Limitations

A limitation when studying hemodynamic effects of carotid stenoses is that patients typically are divided based upon stenosis degree, measured at the carotid bifurcation. The non-stenotic group was added to include less hemodynamically disturbed patients, as a type of controls, since CTA is needed for the territorial analysis and is not clinically motivated in healthy. However, the lack of stenosis in the carotid bifurcations did not exclude the possibility for plaque further up the ICAs and we could not consider them as vascular healthy controls, but as a patient subgroup. A way to bypass this problem was to divide the hemispheres based on flow rate instead of stenosis degree and consider it the threshold for hemodynamic disturbance. In our analysis this perfusion-based division did not yield any differences in CVR either. It is possible that this is an issue of statistical power, which also could explain the lacking inter-territorial difference between ACA and PCA for ipsilateral as well as contralateral hemispheres. However, we have a relatively large group of patients compared to other CFD studies and there were seemingly no extreme differences.

A strength of the study is that we base our boundary conditions on the very same flow rates that we measure, ensuring correct flow rates in the critical area, the circle of Willis. Some model assumptions should however be discussed. For example, MAP was measured prior to the acquisition of the flow rates and ICP was based on reference^32^, which adds potential inaccuracies to the results. Additionally, we assumed MAP at the BA in the posterior models but we consider this assumption to be reasonable due to the exclusion of patients with posterior stenoses. The delay between CTA and MRI scans could also be an issue due to potential changes of the vasculature between scans. However, since we only need to include the larger arteries in our CFD analysis it is unlikely that this would affect the results in any major way, since the larger arteries are not expected to change in size over such a short timeframe. Lastly, verification against in vitro experiments have not been made, but could further support the validity of the methodology presented in the current study. CFD-based modelling of hemodynamics has been widely verified, in e.g. animal models^45^ and in vivo^14,46,47^. Additionally, the 4D flow approach has been validated against 2D-PCMRI (the gold standard method for CBF measurements)^48^. Therefore, in vitro testing would here mainly evaluate the artery segmentation quality. As the ICC was very high, our semi-automated segmentation approach is not very operator-dependent, likely due to the edge detection filters used. However, systematic errors are possible if these filters are too strict or too lenient. Systematic errors in the segmentation, i.e., the size of the arteries, could potentially affect the magnitude of the territorial CVRs, but should not affect the CVR distribution due to all arteries being altered in the same direction if the filters are adjusted.

## Conclusion

This study showcases a new method for assessment of cerebrovascular resistance (CVR) in the major arterial territories of the brain. We present a distribution of CVR of stroke/TIA patients with and without carotid stenoses, identifying values of 34:59:78 mmHg s/ml between the MCA, ACA, and PCA regions. The assessment was carried out with individual arterial trees and flow rates allowing for subject-specific analysis of CVR distribution. No difference in total CVR was found between patients and controls, nor did the method yield any differences in territorial CVR between hemispheres among patients. This could suggest that the presented distribution of territorial CVR may also be representative for the general age group.

## Data Availability

Data is available from the corresponding authors upon reasonable request.
